# Management of Complex Bronchial Ruptures in Blunt Trauma

**DOI:** 10.1186/1749-8090-10-S1-A78

**Published:** 2015-12-16

**Authors:** Hassan Jamal-Eddine, Haisam Saad

**Affiliations:** 1 Department of Thoracic Surgery, Chest Diseases Hospital, Kuwait

## Background/Introduction

Complex bronchial rupture is considered a rare entity. Primary repair is surgically demanding, preferred procedure compared to partial (or total) lung resection that is usually performed in this situation. We present series of complex tracheobronchial injuries reported so far.

## Aims/Objectives

Evaluate the ratio of complex bronchial injuries to simple bronchial injuries and the different management of different complex injuries.

## Method

From 1995-2014, 20 patients were operated for bronchial rupture due to blunt chest trauma. Of these, 7 had complex bronchial injuries (3 located in the right bronchial tree, 3 in the left bronchial tree and 1 had a rupture of both, right and left main bronchi). Injuries to the trachea and simple single bronchial ruptures (within 2.5-3 cm from the carina on the left, and within 1 cm on the right side) were excluded from the study, as well as isolated lobar and segmental injuries. Fiberoptic bronchoscopy was diagnostic in all patients. All 7 patients had primary bronchial repair preserving most of the lung tissue, without need to use a cardiopulmonary bypass.

## Results

All patients survived the procedure. Four patients developed post-operative atelectasis which required bronchoscopy (50%) to remove secretions, one patient had left recurrent laryngeal nerve paralysis (16.6%), one patient required tracheostomy (16.6%). Bronchoscopy follow up at 2 months showed excellent results in all patients (no stenosis or scar formation).

## Discussion/Conclusion

There is an increase in the rate of complex bronchial injuries (7/20 - 35%). Primary repair of this type of bronchial injuries with maximal preservation of the normal functioning lung is possible and preferred. Excellent immediate and long term results are to be expected.

